# Associations of Total Fat and Fatty Acid Intake With the Risk of Type 2 Diabetes Mellitus Among Japanese Adults: Analysis Based on the JACC Study

**DOI:** 10.2188/jea.JE20230076

**Published:** 2024-07-05

**Authors:** Akinori Yaegashi, Takashi Kimura, Kenji Wakai, Hiroyasu Iso, Akiko Tamakoshi

**Affiliations:** 1 Department of Public Health, Graduate School of Medicine, Hokkaido University, Sapporo, Japan; 2 Department of Health and Nutrition, Faculty of Human Science, Hokkaido Bunkyo University, Hokkaido, Japan; 3 Department of Public Health, Faculty of Medicine, Hokkaido University, Sapporo, Japan; 4 Department of Preventive Medicine, Nagoya University, Nagoya, Japan; 5 National Center for Global Health and Medicine, Tokyo, Japan

**Keywords:** fatty acid, Asia, diabetes, Japanese, epidemiology

## Abstract

**Background:**

We prospectively examined the associations of total fat and fatty acid intake with type 2 diabetes (T2D) among Japanese adults.

**Methods:**

This study was conducted using data from the Japan Collaborative Cohort Study for Evaluation of Cancer Risk (JACC). A validated food frequency questionnaire evaluated the intake of total fat and fatty acids. Diabetes was assessed using self-reported data. Multivariable logistic regression analysis was performed to calculate the odds ratios (ORs) and 95% confidence intervals (CIs) of incident T2D across quintiles of total fat and fatty acid intake after adjusting for potential confounders.

**Results:**

A total of 19,088 non-diabetic participants (age range, 40–79 years) enrolled in the JACC between 1988 and 1990 were included in this study. During the 5-year study period, 494 the participants developed T2D. The OR of T2D for the highest versus lowest quintiles was 0.58 (95% CI, 0.37–0.90) for total fat, 0.78 (95% CI, 0.51–1.20) for saturated fatty acid (SFA), 0.55 (95% CI, 0.35–0.86) for monounsaturated fatty acids (MUFA), 0.61 (95% CI, 0.39–0.96) for polyunsaturated fatty acids (PUFA), 0.64 (95% CI, 0.42–0.99) for n-3 PUFA, and 0.70 (95% CI, 0.45–1.09) for n-6 PUFA. Total fat and fatty acid (except SFA and n-6 PUFA) intake were inversely associated with T2D in men. Total fat and fatty acid intake were not associated with T2D in women.

**Conclusion:**

Higher intakes of total fats, MUFA, PUFA, and n-3 PUFA were inversely associated with T2D among Japanese men.

## INTRODUCTION

Type 2 diabetes (T2D) is a metabolic disorder caused by increased blood glucose concentrations due to insufficient insulin secretion and enhanced insulin resistance and increases mortality and morbidity.^1^ The estimated global healthcare cost of diabetes is 673 billion United States dollars.^1^ It is estimated that by 2040, the number of people with diabetes will rise by approximately 55%.^1^ A healthy diet is essential for the prevention of T2D.^2^ In an umbrella review of meta-analyses, various dietary factors, such as total fat and fatty acids, were reported for the prevention of T2D.^3^ For people aged 20 years and older in Japan, the energy intake from total fat is increasing (men 24.3% in 1995, 27.4% in 2019; women 25.9%, 29.2%, respectively). In addition, the intakes of fatty acids other than n-3 PUFA are also increasing (SFA: men 7.2% in 1995, 8.0% in 2019; women 7.9% in 1995, 8.8% in 2019; MUFA: men 7.9% in 2002, 10.4% in 2019; women 8.4% in 2002, 10.8% in 2019; n-6 PUFA: men 4.4% in 2005, 4.9% in 2019; women 4.6% in 2005, 5.2% in 2019; n-3 PUFA: men 1.2% in 2005, 1.1% in 2019; women 1.2% in 2005, 1.2% in 2019).^4^ For people over 20 and over in the United States, the energy intake from total fat, SFA in women, MUFA in women, and PUFA are increasing, and SFA in men and MUFA in men are decreasing (Total fat: men 35.0% in 1989–91 and 36.0% in 2017–2020, women 33.9% in 1989–91 and 37.0% in 2017–2020, SFA: men 12.2% in 1989–91 and 12.0% in 2017–2020, women 11.7% in 1989–91 and 12.0% in 2017–2020, MUFA: men 13.2% in 1989–91 and 12.0% in 2017–2020, women 12.4% in 1989–91 and 13.0% in 2017–2020, PUFA: men 6.9% in 1989–91 and 8.0% in 2017–2020, women 7.1% in 1989–91 and 9.0% in 2017–2020).^5^ Corresponding to increasing intakes of fat and some fatty acids, the number of diabetes cases is rising in both Japan and United States. In Japan, according to the National Health and Nutrition Survey reported by the Ministry of Health, Labour and Welfare, in 2009 and 2019, 15.9% and 19.7% in men and 9.4% and 10.8% in women, respectively, were suspected to have diabetes.^4^ In United States, the age-adjusted prevalence of diagnosed and undiagnosed diabetes was 9.5% in 1999–2002 and 12.0% in 2013–2016.^6^

Previously, some meta-analyses^7^^–^^11^ examined the association of the intake of total fat and some fatty acids with T2D. A recent meta-analysis^11^ study reported that 11 studies were conducted in the United States, 7 studies in Europe, but only 4 studies in Asia, where fat intake is low. The amount of total fat and fatty acids and their sources vary by subject region, which may lead to different results. For example, in Asian populations, the intake of n-3 PUFA was linked to a significant reduction in the incidence of T2D, while in United States populations, it was linked to a significant increase. Thus, more research is needed, especially in Asian countries, such as Japan.

Therefore, the aim of this study was to clarify the associations of total fat and fatty acid intake with the risk of T2D in the Japanese population. We hypothesize that among Japanese, 1) higher n-3 PUFA intakes are associated with lower risk of T2D; 2) higher total fat and fatty acids other than n-3 PUFA intakes are associated with higher risk of T2D.

## METHODS

### Study design and participants

This research was conducted using JACC study data. The protocol for the JACC study has previously been reported.^12^ In brief, the JACC was conducted from 1988 through 1990 and was a multicenter collaborative study in which 24 institutions participated. In 36 of the 45 study areas covered in the JACC, informed consent was obtained from each participant before enrollment into the study. Consent was obtained from the leader of each remaining area as a group. The study protocol followed the guidelines of the Declaration of Helsinki and received approval from the Ethics Committee of Hokkaido University’s Faculty of Medicine (approval number 14-044).

### Assessment of dietary intake

Assessment of dietary intakes, such as total fat and fatty acids, was conducted using a 40-item food frequency questionnaire (FFQ). Participants were asked to report their customary food and drink intake over the past year. The items in the FFQ had for five possible responses to questions on the frequency of consumption: rarely, 1–2 times/month, 1–2 times/week, 3–4 times/week, and almost daily.^13^ As energy intake is strongly correlated with nutrient intake, energy-adjusted dietary intake of nutrients was calculated using the residual method.^14^ A validation study to evaluate dietary intake over a 1-year period using four 3-day weighted dietary records of 85 participants (eight men and 77 women) was used as a reference to estimate size of each meal portion and obtain data on the validity of the FFQ-estimated intakes.^13^ Previously reported, the FFQ-estimated energy-adjusted total fat and fatty acids intakes showed moderate correlation with the ones estimated from dietary records in the validation study. The Spearman rank correlation coefficients were: 0.46 for total fat, 0.50 for SFA, 0.36 for MUFA, 0.15 for PUFA, 0.21 for n-3 PUFA, and 0.16 for n-6 PUFA.^13^ Intake of energy and nutrients, such as total fat and fatty acids, were computed using the Standard Tables of Food Composition in Japan, 5^th^ Revised and Enlarged Edition.^15^

### Assessment of diabetic status

The 5-year cumulative incidence of diabetes was used for analysis because the exact dates of the diabetes diagnoses of the participants were unknown. Therefore, an individual with incident diabetes was a participant who did not have diabetes at baseline but reported physician-diagnosed diabetes by the fifth follow-up year. In a sample 1,230 men and 1,837 women, the diagnosis of diabetes that was self-reported has been confirmed by comparing it with therapy data and laboratory findings.^16^ The sensitivity and specificity were found to be 70% and 95% for men and 75% and 98% for women, respectively. Information on the criteria used to diagnose diabetes has been previously explained.^16^

### Assessment of other factors

The baseline self-administered questionnaire included items on lifestyle factors, including current smoking and drinking habits, hours of exercise, hours spent walking; medical history of diabetes, cancer, or myocardial infarction; family history of diabetes and hypertension; and height and weight. To calculate the body mass index (BMI), self-reported weight in kilograms was divided by the square of self-reported body height in meters.

### Statistical analysis

Statistical analyses were performed using SAS software (version 9.4; SAS Institute Inc., Cary, NC, USA). Intake of six nutrients were examined (total fat, SFA, MUFA, PUFA, n-3 PUFA, and n-6 PUFA). Men and women were analyzed separately because the Japanese dietary intake differs according to sex (men: 2,118 kcal; total fat 66.4 g; women: 1,709 kcal; total fat 56.7 g).^4^

To analyze the relationship between the intake of total fat and fatty acids and various factors, we categorized the intake into five groups. We used the Mantel-Haenszel chi-squared test for categorical variables and linear regression analysis for continuous variables, with the median intake in each quintile category of total fat and fatty acid intake. Multivariable logistic regression was performed to evaluate the association between fat intake and the risk of T2D. The lowest quintile of fat intake was used as the reference group for the analysis. Model 1 was adjusted for age and stratified jointly according to area of residence (Hokkaido, Tohoku, Kanto, Chubu, Kinki, Chugoku, and Kyushu). Model 2 was additionally adjusted for family history of diabetes (yes or no), family history of hypertension (yes or no), smoking status (never, former smoker, or current smoker), BMI (<18.5, 18.5–24.9, 25.0–30.0, or >30.0 kg/m^2^), hours of walking (almost none, daily 0.5, 0.6–0.9, or ≥1.0 h), hours of exercise (almost none, weekly 1–2, 3–4, or ≥5 h), alcohol consumption (never, former drinker, or current drinker), energy intake (kcal, continuous), and carbohydrate (g/day, continuous). An indicator variable for missing data was created for each covariate. In addition, stratified analyses by age (<65 and ≥65 years) among both men and women were conducted. Further stratified analyses according to other factors, such as BMI, were considered but were not performed owing to the low incidence of diabetes among the participants. To calculate the *P* value for linear trend, we used a continuous variable of total fat and fatty acid intake assigning the median values in each quartile. *P* (two-tailed) <0.05 was considered statistically significant.

## RESULTS

**Figure 1.**
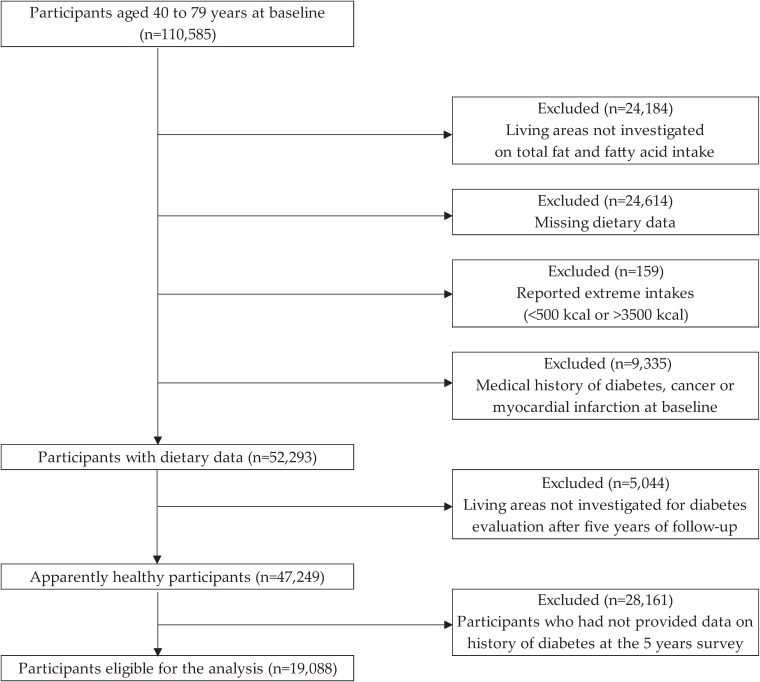
Flow diagram of participant inclusion

In the JACC study, 110,585 participants completed the questionnaire. We excluded those who were from areas not investigated on total fat and fatty acid intake (*n* = 24,184); those with missing dietary data (*n* = 24,614); those who reported extreme energy intakes (<500 kcal or >3,500 kcal) (*n* = 159); those with a medical history of diabetes, cancer, or myocardial infarction at baseline (*n* = 9,335); those living in areas not investigated for the development of diabetes after 5 years of follow-up (*n* = 5,044); and those who did not provide data on diabetes at the fifth year follow-up survey (*n* = 28,161). Thus, a total of 19,088 participants (7,044 men and 12,044 women) were included in the present study (Figure 1).

A total of 494 (2.6%) participants (men, *n* = 247 [3.5%]; women, *n* = 247 [2.0%]) developed T2D during the study period. The baseline characteristics of the study population stratified according to quintiles of energy-adjusted total fat and fatty acid intake are presented in Table 1 and Table 2. For men, participants with higher total fat and fatty acid intake were older, exercised for longer, and had a lower proportion of current drinkers than those with lower total fat and fatty acid intake. For both sexes, participants with higher total fat and fatty acid intake had higher total protein intake, lower carbohydrate intake, and lower proportion of current smokers than those with lower total fat and fatty acid intake.

**Table 1.** Baseline characteristics of participants according to quintiles of energy-adjusted^a^ total fat and fatty acid intake among men

	Total fat			SFA			MUFA		
		
	Q1 (low)	Q3	Q5 (high)	Q1 (low)	Q3	Q5 (high)	Q1 (low)	Q3	Q5 (high)
Age, years	54.8 (9.2)	55.7 (9.4)	56.9 (9.5)^**^	55.1 (9.3)	55.8 (9.5)	56.5 (9.7)^**^	54.9 (9.3)	55.6 (9.5)	56.8 (9.4)^**^
Family history of diabetes, %	7.9	12.5	10.4^**^	8.4	11.3	11.1	8.3	12.6	10.2^**^
Family history of hypertension, %	35.1	36.6	34.1	35.4	36.4	34.4	35.7	37.0	34.0
Current smoker, %	57.4	50.3	47.8^**^	56.5	47.3	51.6^**^	57.1	52.2	48.8^**^
Current drinker, %	85.4	82.9	70.3^**^	84.9	76.2	70.0^**^	84.7	76.9	72.0^**^
BMI, kg/m^2^	22.7 (2.6)	22.8 (4.4)	22.6 (2.6)	22.7 (2.6)	22.8 (4.4)	22.5 (2.6)	22.7 (2.6)	22.6 (2.7)	22.5 (2.7)
BMI ≥25 kg/m^2^, %	18.2	18.6	16.3	17.1	18.6	15.8	17.5	16.6	16.7
Exercise ≥5 h/week, %	6.2	7.3	9.7	7.0	7.1	8.9	6.3	6.7	9.9^**^
Walking ≥0.5 h/week, %	86.7	86.4	89.0	86.1	87.6	89.5	86.7	87.0	89.4^*^
Energy intake, kcal/day	1,920 (516)	1,737 (491)	1,899 (516)	1,883 (526)	1,757 (500)	1,855 (497)	1,929 (516)	1,739 (500)	1,906 (522)
Carbohydrate, % energy	61.3 (11.5)	57.7 (9.5)	53.4 (8.0)^**^	60.5 (11.6)	57.9 (9.6)	54.2 (8.4)^**^	61.4 (11.4)	57.5 (9.5)	53.3 (8.2)^**^
Total protein, % energy	9.6 (1.3)	12.7 (1.5)	15.0 (2.0)^**^	10.0 (1.6)	12.7 (1.9)	14.4 (2.3)^**^	9.8 (1.4)	12.6 (1.7)	14.9 (2.1)^**^
Total fat, % energy	10.4 (1.8)	16.7 (1.9)	22.9 (3.6)^**^	10.8 (2.2)	16.7 (2.6)	22.5 (3.9)^**^	10.6 (1.9)	16.7 (2.1)	22.8 (3.7)^**^
SFA, % energy	3.0 (0.7)	5.1 (1.0)	7.2 (1.6)^**^	2.9 (0.5)	5.0 (0.7)	7.6 (1.5)^**^	3.0 (0.7)	5.1 (1.1)	7.2 (1.6)^**^
MUFA, % energy	3.2 (0.6)	5.3 (0.8)	7.5 (1.4)^**^	3.3 (0.8)	5.3 (1.0)	7.3 (1.4)^**^	3.2 (0.6)	5.3 (0.7)	7.5 (1.3)^**^
PUFA, % energy	2.8 (0.7)	4.1 (0.8)	5.2 (1.0)^**^	3.1 (0.8)	4.1 (1.0)	4.7 (1.2)^**^	2.9 (0.8)	4.0 (0.9)	5.1 (1.0)^**^
n-3 PUFA, % energy	0.5 (0.2)	0.8 (0.2)	1.0 (0.3)^**^	0.6 (0.2)	0.8 (0.3)	0.9 (0.3)^**^	0.5 (0.2)	0.8 (0.2)	1.0 (0.3)^**^
n-6 PUFA, % energy	2.3 (0.6)	3.2 (0.7)	4.1 (0.8)^**^	2.5 (0.7)	3.3 (0.8)	3.8 (0.9)^**^	2.4 (0.7)	3.2 (0.7)	4.1 (0.8)^**^



	PUFA			n-3 PUFA			n-6 PUFA		
		
	Q1 (low)	Q3	Q5 (high)	Q1 (low)	Q3	Q5 (high)	Q1 (low)	Q3	Q5 (high)

Age, years	54.7 (9.2)	55.9 (9.4)	57.2 (9.4)^**^	54.3 (9.4)	55.9 (10.0)	57.3 (9.1)^**^	55.0 (9.2)	55.8 (9.6)	57.4 (9.5)^**^
Family history of diabetes, %	8.5	12.3	9.2^**^	8.8	10.1	9.9	8.4	11.4	8.9
Family history of hypertension, %	34.5	36.1	33.1	35.7	35.9	33.5	34.0	36.7	33.4
Current smoker, %	57.0	51.1	43.9^**^	56.2	51.5	47.3^**^	57.3	51.1	43.9^**^
Current drinker, %	84.3	79.6	70.8^**^	81.3	77.6	76.1^**^	84.7	77.9	69.7^**^
BMI, kg/m^2^	22.6 (2.6)	22.7 (4.3)	22.7 (2.7)	22.6 (2.6)	22.6 (2.6)	22.8 (4.4)	22.6 (2.6)	22.7 (4.4)	22.6 (2.7)
BMI ≥25 kg/m^2^, %	17.9	16	18.3	18	16.3	18.5	17.4	16.6	17.8
Exercise ≥5 h/week, %	7.2	7.0	10.3^**^	6.4	6.8	9.5^**^	7.3	7.3	10.4^**^
Walking ≥0.5 h/week, %	86.6	87.6	88.4^**^	87.1	85.9	87.9^**^	86.4	87.1	88.4^**^
Energy intake, kcal/day	1,922 (491)	1,739 (494)	1,904 (552)	1,905 (477)	1,738 (508)	1,890 (520)	1,925 (490)	1,735 (495)	1,895 (547)
Carbohydrate, % energy	60.6 (11.5)	57.3 (9.6)	54.8 (8.3)^**^	61.8 (10.9)	57.5 (9.1)	53.4 (8.6)^**^	60.0 (11.6)	57.6 (9.7)	55.3 (8.3)^**^
Total protein, % energy	9.8 (1.5)	12.6 (1.5)	15.1 (2.0)^**^	9.7 (1.4)	12.6 (1.4)	15.2 (2.0)^**^	10.0 (1.7)	12.6 (1.7)	14.9 (2.1)^**^
Total fat, % energy	11.8 (3.3)	16.8 (3.5)	21.5 (4.2)^**^	12.4 (3.8)	17.0 (4.0)	20.6 (4.4)^**^	12.0 (3.4)	16.7 (3.5)	21.5 (4.3)^**^
SFA, % energy	3.9 (1.6)	5.2 (1.7)	6.2 (1.7)^**^	4.1 (1.8)	5.2 (1.8)	5.9 (1.7)^**^	3.9 (1.6)	5.1 (1.7)	6.2 (1.7)^**^
MUFA, % energy	3.7 (1.2)	5.3 (1.3)	6.9 (1.6)^**^	3.9 (1.3)	5.4 (1.5)	6.6 (1.6)^**^	3.8 (1.2)	5.3 (1.3)	6.9 (1.6)^**^
PUFA, % energy	2.5 (0.4)	4.0 (0.3)	5.6 (0.8)^**^	2.8 (0.6)	4.1 (0.7)	5.2 (1.0)^**^	2.6 (0.5)	4.0 (0.3)	5.6 (0.8)^**^
n-3 PUFA, % energy	0.5 (0.2)	0.8 (0.2)	1.1 (0.2)^**^	0.4 (0.1)	0.8 (0.1)	1.2 (0.2)^**^	0.5 (0.2)	0.8 (0.2)	1.0 (0.2)^**^
n-6 PUFA, % energy	2.0 (0.4)	3.2 (0.3)	4.5 (0.7)^**^	2.3 (0.6)	3.3 (0.7)	4.0 (0.9)^**^	2.0 (0.4)	3.2 (0.2)	4.5 (0.6)^**^

BMI, body mass index; MUFA, monounsaturated fatty acid; PUFA, polyunsaturated fatty acid; SD, standard deviation; SFA, saturated fatty acid.

All values are mean (SD) or percentages.

^a^Total fat and fatty acid levels were adjusted for energy intake using the nutrient residual model.

*P*-trend was calculated using the Mantel-Haenszel chi-squared test for categorical variables and linear regression analysis for continuous variables, with the median intake in each quintile category of total fat and fatty acid intake.

^*^<0.05, ^**^<0.01.

Participants with missing information were excluded (BMI: *n* = 206; smoking status: *n* = 117; drinking status: *n* = 0; sports information: *n* = 134; walking information: *n* = 181).

**Table 2.** Baseline characteristics of participants according to quintiles of energy-adjusted^a^ total fat and fatty acid intake among women

	Total fat			SFA			MUFA		
		
	Q1 (low)	Q3	Q5 (high)	Q1 (low)	Q3	Q5 (high)	Q1 (low)	Q3	Q5 (high)
Age, years	57.0 (9.6)	55.1 (9.3)	54.6 (9.2)^**^	57.5 (9.4)	55.6 (9.3)	54.3 (9.3)^**^	57.3 (9.5)	55.2 (9.3)	54.4 (9.2)^**^
Family history of diabetes, %	9.7	11.0	11.0	9.5	11.0	12.1^*^	10.3	10.8	11.0
Family history of hypertension, %	33.1	36.3	36.6^**^	32.8	37.2	37.2^**^	33.1	37.7	36.4^**^
Current smoker, %	5.6	3.0	2.8^**^	4.8	2.7	3.5^**^	5.1	2.9	2.8^**^
Current drinker, %	25.2	24.5	25.7	23.2	23.6	28.9^**^	24.5	23.5	26.4
BMI, kg/m^2^	23.0 (6.4)	22.9 (2.9)	22.6 (2.8)	23.1 (6.4)	22.9 (2.9)	22.5 (2.7)^**^	22.9 (3.1)	22.9 (6.2)	22.6 (2.8)^**^
BMI ≥25 kg/m^2^, %	22.6	20.9	18.1	23.5	21.1	16.0	21.7	20.5	17.7
Exercise ≥5 h/week, %	4.6	3.3	5.5^**^	5.2	3.8	4.8	4.9	3.7	5.1^*^
Walking ≥0.5 h/week, %	87.4	86.1	87.8^**^	87.5	87.4	89.2^**^	87.7	86.6	88.0^**^
Energy intake, kcal/day	1,544 (422)	1,378 (323)	1,546 (368)	1,526 (412)	1,401 (341)	1,520 (357)	1,544 (414)	1,383 (332)	1,542 (371)
Carbohydrate, % energy	71.2 (6.1)	62.5 (3.7)	54.8 (4.4)^**^	70.4 (6.2)	62.5 (4.5)	56.0 (5.0)^**^	71.1 (6.0)	62.5 (3.9)	55.0 (4.5)^**^
Total protein, % energy	11.9 (1.5)	15.0 (1.5)	17.0 (1.9)^**^	12.5 (1.9)	15.0 (1.9)	16.3 (2.1)^**^	12.1 (1.7)	15.0 (1.7)	16.8 (2.0)^**^
Total fat, % energy	13.7 (2.4)	20.7 (1.1)	27.0 (2.8)^**^	14.2 (2.9)	20.7 (2.4)	26.3 (3.2)^**^	13.9 (2.6)	20.7 (1.5)	26.8 (2.9)^**^
SFA, % energy	4.0 (0.9)	6.4 (0.9)	8.6 (1.4)^**^	3.8 (0.7)	6.4 (0.4)	8.9 (1.2)^**^	4.1 (1.0)	6.4 (1.0)	8.5 (1.5)^**^
MUFA, % energy	4.2 (0.9)	6.6 (0.6)	8.9 (1.1)^**^	4.4 (1.0)	6.6 (1.0)	8.6 (1.3)^**^	4.2 (0.8)	6.6 (0.4)	9.0 (1.1)^**^
PUFA, % energy	3.5 (0.8)	4.8 (0.8)	5.8 (1.0)^**^	3.9 (1.1)	4.8 (1.1)	5.2 (1.1)^**^	3.6 (0.9)	4.8 (0.9)	5.8 (1.0)^**^
n-3 PUFA, % energy	0.7 (0.2)	1.0 (0.3)	1.2 (0.3)^**^	0.8 (0.3)	1.0 (0.3)	1.0 (0.3)^**^	0.7 (0.2)	1.0 (0.3)	1.1 (0.3)^**^
n-6 PUFA, % energy	2.8 (0.7)	3.8 (0.7)	4.7 (0.8)^**^	3.1 (0.9)	3.9 (0.9)	4.2 (0.9)^**^	2.9 (0.8)	3.8 (0.7)	4.6 (0.8)^**^



	PUFA			n-3 PUFA			n-6 PUFA		
		
	Q1 (low)	Q3	Q5 (high)	Q1 (low)	Q3	Q5 (high)	Q1 (low)	Q3	Q5 (high)

Age, years	56.3 (9.7)	55.0 (9.4)	55.9 (9.1)	55.6 (9.8)	55.0 (9.4)	56.4 (8.9)^*^	56.5 (9.6)	55.0 (9.4)	55.9 (9.1)^*^
Family history of diabetes, %	11.2	11.9	9.3^**^	12.0	11.8	9.2	11.0	12.1	10.1^**^
Family history of hypertension, %	33.9	36.9	36.5^*^	34.8	37.8	37.0	34.7	36.7	36.8
Current smoker, %	6.5	3.5	1.7^**^	5.7	3.9	1.9^**^	6.4	3.3	1.8^**^
Current drinker, %	29.5	24.6	20.8^**^	30.4	23.4	22.4^**^	30.0	24.7	21.0^**^
BMI, kg/m^2^	22.9 (6.3)	22.8 (2.9)	22.9 (2.9)	22.7 (3.0)	22.8 (2.9)	23.0 (3.0)^**^	22.9 (6.3)	22.7 (2.9)	22.9 (2.9)
BMI ≥25 kg/m^2^, %	21.4	19.0	22.2	19.7	20.5	23.0	21.9	18.9	21.9
Exercise ≥5 h/week, %	4.6	3.5	5.1^**^	3.8	4.0	5.7^**^	4.8	3.5	5.2^**^
Walking ≥0.5 h/week, %	89.2	86.9	86.2^**^	88.1	88.4	85.6^**^	89.3	87.2	86.7^**^
Energy intake, kcal/day	1,529 (396)	1,385 (333)	1,547 (384)^**^	1,533 (383)	1,393 (347)	1,521 (375)^**^	1,525 (396)	1,384 (331)	1,544 (385)^**^
Carbohydrate, % energy	69.5 (7.1)	62.6 (5.2)	56.7 (5.3)^**^	68.7 (7.0)	62.7 (5.6)	57.0 (5.6)^**^	69.0 (7.5)	62.5 (5.4)	57.1 (5.5)^**^
Total protein, % energy	12.0 (1.6)	14.9 (1.4)	17.2 (1.9)^**^	12.0 (1.5)	14.8 (1.2)	17.5 (1.8)^**^	12.3 (1.9)	14.9 (1.7)	16.8 (1.9)^**^
Total fat, % energy	15.2 (3.8)	20.8 (3.6)	25.0 (3.7)^**^	16.5 (4.7)	20.7 (4.0)	24.1 (4.0)^**^	15.4 (4.0)	20.9 (3.6)	24.9 (3.8)^**^
SFA, % energy	5.1 (1.8)	6.5 (1.8)	7.2 (1.6)^**^	5.6 (2.1)	6.5 (1.8)	6.9 (1.6)^**^	5.1 (1.8)	6.6 (1.8)	7.2 (1.6)^**^
MUFA, % energy	4.9 (1.4)	6.7 (1.4)	8.1 (1.5)^**^	5.3 (1.7)	6.7 (1.5)	7.8 (1.6)^**^	4.9 (1.4)	6.7 (1.4)	8.1 (1.5)^**^
PUFA, % energy	3.2 (0.5)	4.7 (0.2)	6.3 (0.7)^**^	3.5 (0.8)	4.8 (0.8)	6.0 (0.9)^**^	3.2 (0.6)	4.7 (0.3)	6.3 (0.7)^**^
n-3 PUFA, % energy	0.6 (0.2)	0.9 (0.2)	1.3 (0.3)^**^	0.6 (0.1)	0.9 (0.1)	1.4 (0.2)^**^	0.7 (0.3)	0.9 (0.3)	1.2 (0.3)^**^
n-6 PUFA, % energy	2.5 (0.5)	3.8 (0.3)	5.1 (0.6)^**^	2.9 (0.7)	3.9 (0.7)	4.5 (0.9)^**^	2.5 (0.4)	3.8 (0.2)	5.1 (0.6)^**^

BMI, body mass index; MUFA, monounsaturated fatty acid; PUFA, polyunsaturated fatty acid; SD, standard deviation; SFA, saturated fatty acid.

All values are mean (SD) or percentages.

^a^Total fat and fatty acid levels were adjusted for energy intake using the nutrient residual model.

*P*-trend was calculated using the Mantel-Haenszel chi-squared test for categorical variables and linear regression analysis for continuous variables, with the median intake in each quintile category of total fat and fatty acid intake.

^*^<0.05, ^**^<0.01.

Participants with missing information were excluded (BMI: *n* = 408; smoking status: *n* = 928; drinking status: *n* = 462; sports information: *n* = 338; walking information: *n* = 430).

The odds ratios (ORs) and 95% confidence intervals (CIs) of T2D stratified according to the energy-adjusted total fat and fatty acid intake in men and women are shown in Table 3 and Table 4. For men, the multivariable-adjusted OR of incident T2D for the highest versus lowest quintiles was 0.58 (95% CI, 0.37–0.90; *P*-trend = 0.037) for total fat, 0.78 (95% CI, 0.51–1.21; *P*-trend = 0.120) for SFA, 0.55 (95% CI, 0.35–0.86; *P*-trend = 0.019) for MUFA, 0.61 (95% CI, 0.39–0.96; *P*-trend = 0.059) for PUFA, 0.64 (95% CI, 0.42–0.99; *P*-trend = 0.111) for n-3 PUFA, and 0.70 (95% CI, 0.45–1.09; *P*-trend = 0.115) for n-6 PUFA, indicating that total fat, MUFA, PUFA, and n-3 PUFA were inversely associated with T2D. For women, total fat and fatty acid intake were not significantly associated with T2D.

**Table 3.** Odds ratios and 95% confidence intervals of type 2 diabetes according to quintiles of energy-adjusted^a^ total fat and fatty acid intake among men

	Q1 (low)	Q2	Q3	Q4	Q5 (high)	*P* for trend^b^
Total fat, g/day	<25.34	25.34–30.31	30.32–34.45	34.46–40.05	>40.05	
Number of participants	1,408	1,409	1,409	1,409	1,409	
Number of cases	61	44	50	51	41	
Model 1	1.00 (reference)	0.72 (0.48–1.07)	0.82 (0.56–1.21)	0.81 (0.55–1.20)	**0.66 (0.43–0.995)**	0.102
Model 2	1.00 (reference)	0.72 (0.48–1.08)	0.78 (0.52–1.17)	0.79 (0.53–1.19)	**0.58 (0.37–0.90)**	**0.037**
SFA, g/day	<7.12	7.12–8.90	8.91–10.50	10.51–12.57	>12.57	
Number of participants	1,408	1,409	1,409	1,409	1,409	
Number of cases	52	51	57	39	48	
Model 1	1.00 (reference)	1.00 (0.67–1.49)	1.11 (0.75–1.63)	0.73 (0.48–1.13)	0.88 (0.59–1.33)	0.289
Model 2	1.00 (reference)	0.98 (0.66–1.47)	1.07 (0.72–1.59)	0.70 (0.45–1.08)	0.78 (0.51–1.21)	0.120
MUFA, g/day	<7.79	7.79–9.42	9.43–10.94	10.95–12.89	>12.89	
Number of participants	1,408	1,409	1,409	1,409	1,409	
Number of cases	58	50	45	55	39	
Model 1	1.00 (reference)	0.86 (0.58–1.27)	0.77 (0.52–1.15)	0.92 (0.63–1.36)	**0.65 (0.43–0.99)**	0.084
Model 2	1.00 (reference)	0.83 (0.56–1.24)	0.74 (0.49–1.11)	0.86 (0.58–1.28)	**0.55 (0.35–0.86)**	**0.019**
PUFA, g/day	<6.19	6.19–7.40	7.41–8.45	8.46–9.73	>9.73	
Number of participants	1,408	1,409	1,409	1,409	1,409	
Number of cases	66	47	44	50	40	
Model 1	1.00 (reference)	0.71 (0.48–1.05)	**0.66 (0.44–0.99)**	0.78 (0.52–1.16)	**0.62 (0.40–0.96)**	0.061
Model 2	1.00 (reference)	0.72 (0.48–1.07)	0.66 (0.44–1.00)	0.77 (0.51–1.16)	**0.61 (0.39–0.96)**	0.059
n-3 PUFA, g/day	<1.13	1.13–1.38	1.39–1.65	1.66–1.99	>1.99	
Number of participants	1,408	1,409	1,409	1,409	1,409	
Number of cases	63	38	55	46	45	
Model 1	1.00 (reference)	**0.60 (0.40–0.91)**	0.88 (0.60–1.29)	0.74 (0.50–1.10)	0.73 (0.49–1.10)	0.282
Model 2	1.00 (reference)	**0.59 (0.39–0.90)**	0.87 (0.59–1.28)	0.70 (0.46–1.06)	**0.64 (0.42–0.99)**	0.111
n-6 PUFA, g/day	<4.95	4.95–5.94	5.95–6.80	6.81–7.84	>7.84	
Number of participants	1,408	1,409	1,409	1,409	1,409	
Number of cases	49	45	52	47	54	
Model 1	1.00 (reference)	**0.64 (0.43–0.95)**	0.79 (0.54–1.17)	**0.59 (0.39–0.91)**	0.69 (0.45–1.05)	0.088
Model 2	1.00 (reference)	**0.65 (0.43–0.98)**	0.80 (0.54–1.20)	**0.60 (0.38–0.92)**	0.70 (0.45–1.09)	0.115

BMI, body mass index; MUFA, monounsaturated fatty acid; PUFA, polyunsaturated fatty acid, Q, quintile; SFA, saturated fatty acid.

Bold *P* values are statistically significant (*P* < 0.05).

The lowest quintiles of total fat and fatty acid intake were used as the reference group in the analysis. Model 1 was adjusted for age and stratified jointly according to areas (Hokkaido, Tohoku, Kanto, Chubu, Kinki, Chugoku, and Kyusyu). Model 2 was additionally adjusted for family history of diabetes (yes, no); family history of hypertension (yes, no); smoking status (never, former smoker, current smoker); body mass index (<18.5, 18.5–24.9, 25.0–30.0, >30.0 kg/m^2^); walking hours (almost none, daily 0.5, 0.6–0.9, and ≥1.0 h); hours of exercise (almost none, weekly 1–2, 3–4, and ≥5 h); alcohol consumption habit (never, former drinker, current drinker); energy intake (kcal, continuous); and carbohydrate (g/day; continuous).

^a^Total fat and fatty acid intake were adjusted for energy intake using the nutrient residual model.

^b^The *P* value for linear trend was calculated using a continuous variable of total fat and fatty acid intake assigning the median values in each quartile.

**Table 4.** Odds ratios and 95% confidence intervals of type 2 diabetes according to quintile categories of energy-adjusted^a^ total fat and fatty acid intake among women

Women	Q1 (low)	Q2	Q3	Q4	Q5 (high)	*P* for trend^b^
Total fat, g/day	<26.75	26.75–31.06	31.07–34.78	34.79–39.25	>39.25	
Number of participants	2,408	2,409	2,409	2,409	2,409	
Number of cases	61	47	47	41	46	
Model 1	1.00 (reference)	0.76 (0.51–1.12)	0.77 (0.52–1.14)	0.67 (0.44–1.01)	0.76 (0.51–1.14)	0.144
Model 2	1.00 (reference)	0.72 (0.47–1.11)	0.76 (0.48–1.21)	0.70 (0.41–1.18)	0.80 (0.43–1.51)	0.455
SFA, g/day	<7.68	7.68–9.36	9.37–10.83	10.84–12.53	>12.53	
Number of participants	2,408	2,409	2,409	2,409	2,409	
Number of cases	53	58	43	44	44	
Model 1	1.00 (reference)	1.11 (0.76–1.62)	0.82 (0.54–1.24)	0.85 (0.56–1.29)	0.82 (0.54–1.25)	0.182
Model 2	1.00 (reference)	1.12 (0.75–1.69)	0.85 (0.53–1.34)	0.93 (0.57–1.54)	0.93 (0.53–1.65)	0.643
MUFA, g/day	<8.34	8.34–9.85	9.86–11.19	11.20–12.82	>12.82	
Number of participants	2,408	2,409	2,409	2,409	2,409	
Number of cases	59	55	40	46	42	
Model 1	1.00 (reference)	0.95 (0.65–1.38)	0.68 (0.45–1.03)	0.78 (0.53–1.17)	0.73 (0.49–1.11)	0.084
Model 2	1.00 (reference)	0.86 (0.57–1.30)	0.65 (0.40–1.04)	0.73 (0.45–1.21)	0.68 (0.37–1.25)	0.181
PUFA, g/day	<6.16	6.16–7.17	7.18–8.06	8.07–9.15	>9.15	
Number of participants	2,408	2,409	2,409	2,409	2,409	
Number of cases	54	46	47	54	41	
Model 1	1.00 (reference)	0.85 (0.57–1.28)	0.90 (0.60–1.36)	1.05 (0.70–1.57)	0.80 (0.51–1.25)	0.585
Model 2	1.00 (reference)	0.87 (0.57–1.34)	0.96 (0.62–1.51)	1.16 (0.72–1.85)	0.91 (0.51–1.60)	0.899
n-3 PUFA, g/day	<1.11	1.11–1.35	1.36–1.60	1.61–1.91	>1.91	
Number of participants	2,408	2,409	2,409	2,409	2,409	
Number of cases	55	44	48	54	41	
Model 1	1.00 (reference)	0.81 (0.54–1.22)	0.93 (0.62–1.38)	1.05 (0.71–1.55)	0.78 (0.51–1.20)	0.557
Model 2	1.00 (reference)	0.80 (0.52–1.22)	0.88 (0.58–1.36)	1.05 (0.67–1.63)	0.75 (0.45–1.28)	0.608
n-6 PUFA, g/day	<4.90	4.90–5.74	5.75–6.46	6.47–7.32	>7.32	
Number of participants	2,408	2,409	2,409	2,409	2,409	
Number of cases	57	47	43	47	48	
Model 1	1.00 (reference)	0.83 (0.55–1.23)	0.77 (0.51–1.17)	0.85 (0.56–1.29)	0.88 (0.57–1.35)	0.617
Model 2	1.00 (reference)	0.86 (0.57–1.31)	0.83 (0.53–1.29)	0.95 (0.60–1.51)	1.01 (0.60–1.70)	0.889

BMI, body mass index; MUFA, monounsaturated fatty acid; PUFA, polyunsaturated fatty acid, Q, quintile; SFA, saturated fatty acid.

Bold *P* values are statistically significant (*P* < 0.05).

The lowest quartiles of total fat and fatty acid intake were used as the reference group in the analysis. Model 1 was adjusted for age and stratified jointly according to areas (Hokkaido, Tohoku, Kanto, Chubu, Kinki, Chugoku, and Kyusyu). Model 2 was additionally adjusted for family history of diabetes (yes, no); family history of hypertension (yes, no); smoking status (never, former smoker, current smoker); body mass index (<18.5, 18.5–24.9, 25.0–30.0, >30.0 kg/m^2^); walking hours (almost none, daily 0.5, 0.6–0.9, and ≥1.0 h); hours of exercise (almost none, weekly 1–2, 3–4, and ≥5 h); alcohol consumption habit (never, former drinker, current drinker); energy intake (kcal, continuous); and carbohydrate (g/day; continuous).

^a^Total fat and fatty acid intake were adjusted for energy intake using the nutrient residual model.

^b^The *P* value for linear trend was calculated using a continuous variable of total fat and fatty acid intake assigning the median values in each quartile.

eTable 1 and eTable 2 show results of stratified analyses by age (<65 and ≥65 years) among both men and women. In men, higher MUFA and n-3 PUFA intake were inversely associated with T2D among those aged <65 years, while the association was not significant among those aged ≥65 years. Among those aged ≥65 years, consumption of more PUFA in men and consumption of more n-6 PUFA in women were inversely associated with T2D. However, this association was not observed among those aged <65 years.

## DISCUSSION

In this study, we analyzed the associations of total fat and fatty acid intake with the risk of T2D in Japanese adults. The results of this study indicated that total fat, MUFA, PUFA, and n-3 PUFA intakes were inversely associated with T2D in Japanese men but not in women. SFA and n-6 PUFA intake were not associated with T2D in either sex. In women, total fat and fatty acid intake were not significantly associated with T2D. Given the large difference in ORs for the associations of total fat, MUFA, PUFA, and n-3 PUFA with T2D in the Q1 and Q2–5 group of men and the fact that OR of Q1–Q5 is not clearly linear, we think that the lowest total fat, MUFA, PUFA, and n-3 PUFA intake in men may be at a risk of T2D, but higher intake itself is not associated with a reduced risk.

In Japan, the number of diabetes cases is rising, and intakes of total fat and fatty acids other than n-3 PUFA are also increasing. However, our result reported higher intakes of total fat and all fatty acids were not associated with higher risk of T2D. Thus, total fat and fatty acids intake may not be a significant contributor to the incidence of T2D in Japan.

The results of the present study regarding total fat, PUFA, and n-3 PUFA intakes in men are inconsistent with those of previous meta-analyses.^7^^–^^11^ However, most of the studies included in those meta-analyses were not conducted in Asia. In contrast, the results of two studies conducted in Asia,^17^^,^^18^ which were included in the abovementioned meta-analyses,^7^^,^^8^ are consistent with the results of the present study. This discrepancy may be attributed to the fact that Asians have a lower fat intake^19^ and a higher intake of fish rich in n-3 PUFA and n-3 PUFA than non-Asians^20^ (fish intake in 2013: Japan, 48.60 kg/capita/year; United States, 21.51 kg/capita/year; United Kingdom, 20.76 kg/capita/year; n-3 PUFA: Japan, 1.3% energy in men, 1.4% energy in women; United States, 0.7% energy in men, 0.7% energy in women; United Kingdom, 0.7% energy in men, 0.8% energy in women). Although it is believed that n-3 PUFA may reduce the risk of developing diabetes by modulating insulin sensitivity in phospholipid membranes,^20^ the detailed reason for the discrepancy of association of n-3 PUFA and T2D between Asia and non-Asia is unknown.

A previous cross-sectional study demonstrated a favorable relationship between MUFA and β-cell insulin secretion.^21^ In addition, a cohort study indicated that increased intake of MUFA is associated with better β-cell function.^22^ This is because intake of MUFA preserves or even enhances β-cell proliferation and has as an anti-apoptotic effect.^23^ The results of the present study indicated that MUFA intake is associated with a reduced risk of T2D in men, a finding that is in line with those of the abovementioned previous studies.^21^^–^^23^ In contrast, a previous meta-analysis^11^ indicated that MUFA intake is not associated with the risk of T2D. The reason for the discrepancy between the results of the present study and those of this previous meta-analysis^11^ are unknown. Therefore, further research is needed clarify the relationship between MUFA intake and the risk of T2D.

The results of the present study for both sexes indicated that n-6 PUFA and SFA intake were not associated with the risk of T2D, a finding that is consistent with those of a previous meta-analysis.^11^ Experimental evidence suggests that n-6 PUFA is associated with favorable insulin sensitivity and protection against the development of T2D.^24^ In contrast, in vitro experimental data have demonstrated that SFA induces insulin resistance.^25^ Thus, although n-6 PUFA and SFA have mechanisms associated with the reduction or increase in the risk of developing diabetes, they may not affect the risk of diabetes in the amounts habitually consumed by humans.

The results of the present study indicate that there are sex-specific differences in the associations of total fat intake and intake of most fatty acids with the risk of T2D. Total fat, MUFA, PUFA, and n-3 PUFA intakes were associated with a lower risk of T2D in men but not in women. These sex-specific differences may be attributed to the fact that women tend to follow a healthier lifestyle in general, and the intake of fat and fatty acids they consumed may not have had a significant impact on their likelihood of developing diabetes.^26^

The present study has several limitations. First, some misclassification of the diagnosis of T2D was unavoidable because all diagnoses were self-reported. However, the self-reported T2D diagnoses were validated, and the results showed moderate sensitivity (70% for men and 75% for women) and specificity (95% for men and 98% for women).^16^ Second, it is worth noting that dietary intakes were only measured once, which means the data may not have fully captured long-term intake. Unfortunately, this could have resulted in an underestimation of the association between dietary intake and the risk of T2D, due to non-differential misclassifications. Third, even though the present study had a prospective design, there is a concern about reverse causation due to the short follow-up period of 5 years. Some participants may have already had prediabetes at the beginning of the study and altered their diet accordingly. Furthermore, we were unable to exclude participants who had undiagnosed diabetes or prediabetes because we did not gather information on their blood glucose levels at the start of the study. Thus, the incidence of T2D in our study may be substituted for prevalence. Fourth, result of this study in the late 1980s may not be applicable to the Japanese population in 2023 because mean ratio of energy intake from total fat are increasing for people 20 and over in Japan (men 24.3% in 1995, 27.4% in 2019; women 25.9% in 1995, 29.2% in 2019).^4^ Fifth, the validation study showed low Spearman rank correlation coefficients of 0.15 for PUFA, 0.21 for n-3 PUFA, and 0.16 for n-6 PUFA, indicating the possibility of misclassification. Sixth, data was only collected from one-third of the eligible participants due to limitations in the 5-year follow-up survey, which was conducted in specific baseline study areas and not all of them. However, there were no significant difference in the characteristics of the participants, including age, BMI, and other factors, between those who responded to the 5-year survey and those who did not.^27^ Finally, our study cannot rule out the possibility of residual confounding factors.

### Conclusion

Higher intakes of total fats, MUFA, PUFA, and n-3 PUFA were inversely associated with T2D among Japanese men. In women, total fat and fatty acid were not significantly associated with T2D.

## SUPPLEMENTARY MATERIAL

eTable 1. Odds ratios and 95% confidence intervals of type 2 diabetes according to quintiles of energy-adjusted^a^ total fat and fatty acid intake by the age (<65 and ≥65 years) among men

eTable 2. Odds ratios and 95% confidence intervals of type 2 diabetes according to quintiles of energy-adjusted^a^ total fat and fatty acid intake by the age (<65 and ≥65 years) among women
