# Supplementary material for: Associations of Total Fat and Fatty Acid Intake With the Risk of Type 2 Diabetes Mellitus Among Japanese Adults: Analysis Based on the JACC Study
Source: J Epidemiol. 2024 Jul 5;34(7):316–23. doi: 10.2188/jea.JE20230076 (PMC11167261; doi:10.2188/jea.JE20230076)
Supplement: Supplementary file 1 [file je-34-316-s001.pdf]

eTable 1. Odds ratios and 95% confidence intervals of type 2 diabetes according to quintiles of energy-adjusted[a] total fat and fatty acid intake by the age (<65 and ≥65 years) among men

	Q1 (low)	Q2	Q3	Q4	Q5 (high)	***P* for trend**[b]
Total fat, g/day						
<65						
Number of participants	1,204	1,179	1,168	1,108	1,088	
Number of cases	51	36	39	32	31	
Model 1	1.00 (reference)	0.75 (0.48–1.16)	0.81 (0.53–1.25)	0.70 (0.44–1.11)	0.69 (0.43–1.10)	0.119
Model 2	1.00 (reference)	0.75 (0.48–1.18)	0.76 (0.48–1.18)	0.69 (0.43–1.10)	0.61 (0.37–1.01)	0.055
≥65						
Number of participants	204	230	241	301	321	
Number of cases	10	8	11	19	10	
Model 1	1.00 (reference)	0.64 (0.25–1.69)	0.88 (0.36–2.15)	1.20 (0.52–2.73)	0.57 (0.23–1.45)	0.529
Model 2	1.00 (reference)	0.59 (0.22–1.61)	0.86 (0.34–2.19)	1.13 (0.47–2.69)	0.47 (0.17–1.31)	0.396
SFA, g/day						
<65						
Number of participants	1,188	1,182	1,155	1,121	1,101	
Number of cases	44	42	45	23	35	
Model 1	1.00 (reference)	0.98 (0.64–1.52)	1.09 (0.71–1.68)	**0.56 (0.33–0.93)**	0.85 (0.53–1.34)	0.169
Model 2	1.00 (reference)	0.93 (0.60–1.44)	1.03 (0.66–1.60)	**0.51 (0.30–0.87)**	0.74 (0.46–1.21)	0.073
≥65						
Number of participants	220	227	254	288	308	
Number of cases	8	9	12	16	13	
Model 1	1.00 (reference)	1.05 (0.39–2.79)	1.28 (0.51–3.23)	1.49 (0.61–3.61)	1.08 (0.43–2.73)	0.772
Model 2	1.00 (reference)	1.24 (0.46–3.37)	1.46 (0.56–3.81)	1.57 (0.63–3.92)	1.10 (0.41–2.96)	0.867
MUFA, g/day						
<65						
Number of participants	1,197	1,179	1,167	1,109	1,095	
Number of cases	49	40	34	37	29	
Model 1	1.00 (reference)	0.84 (0.55–1.30)	0.72 (0.46–1.13)	0.83 (0.54–1.30)	0.65 (0.40–1.04)	0.086
Model 2	1.00 (reference)	0.83 (0.54–1.29)	0.67 (0.42–1.07)	0.77 (0.49–1.22)	**0.55 (0.33–0.92)**	**0.025**
≥65						
Number of participants	211	230	242	300	314	
Number of cases	9	10	11	18	10	
Model 1	1.00 (reference)	0.95 (0.37–2.41)	1.03 (0.41–2.56)	1.33 (0.57–3.09)	0.68 (0.27–1.76)	0.574
Model 2	1.00 (reference)	0.81 (0.31–2.15)	1.01 (0.39–2.62)	1.19 (0.48–2.92)	0.56 (0.20–1.57)	0.410

BMI, body mass index; MUFA, monounsaturated fatty acid; PUFA, polyunsaturated fatty acid, Q, quintile; SFA, saturated fatty acid.
Bold *P* values are statistically significant ($P<0.05$).
The lowest quintiles of total fat and fatty acid intake were used as the reference group in the analysis. Model 1 was adjusted for age and stratified jointly according to areas (Hokkaido, Tohoku, Kanto, Chubu, Kinki, Chugoku, and Kyusyu). Model 2 was additionally adjusted for family history of diabetes (yes, no); family history of hypertension (yes, no); smoking status (never, former smoker, current smoker); body mass index (<18.5, 18.5–24.9, 25.0–30.0, >30.0 kg/m^2); walking hours (almost none, daily 0.5, 0.6–0.9, and ≥1.0 h); hours of exercise (almost none, weekly 1–2, 3–4, and ≥5 h); alcohol consumption habit (never, former drinker, current drinker); energy intake (kcal, continuous); and carbohydrate (g/day; continuous).
[a]Total fat and fatty acid intake were adjusted for energy intake using the nutrient residual model.
[b]The *P* value for linear trend was calculated using a continuous variable of total fat and fatty acid intake assigning the median values in each quartile.

eTable 1. Odds ratios and 95% confidence intervals of type 2 diabetes according to quintiles of energy-adjusted[a] total fat and fatty acid intake by the age (<65 and ≥65 years) among men (continued)

	Q1 (low)	Q2	Q3	Q4	Q5 (high)	***P* for trend[b]**
PUFA, g/day						
<65						
Number of participants	1,212	1,187	1,150	1,121	1,077	
Number of cases	52	41	33	34	29	
Model 1	1.00 (reference)	0.84 (0.55–1.29)	0.71 (0.45–1.12)	0.79 (0.49–1.25)	0.70 (0.43–1.15)	0.145
Model 2	1.00 (reference)	0.87 (0.56–1.35)	0.73 (0.45–1.17)	0.79 (0.49–1.28)	0.68 (0.40–1.14)	0.131
≥65						
Number of participants	196	222	259	288	332	
Number of cases	14	6	11	16	11	
Model 1	1.00 (reference)	**0.32 (0.12–0.86)**	0.49 (0.21–1.15)	0.64 (0.28–1.46)	**0.38 (0.15–0.96)**	0.185
Model 2	1.00 (reference)	**0.28 (0.10–0.78)**	**0.40 (0.16–0.99)**	0.54 (0.23–1.27)	**0.34 (0.13–0.92)**	0.167
n-3 PUFA, g/day						
<65						
Number of participants	1,222	1,180	1,114	1,134	1,097	
Number of cases	50	32	42	38	27	
Model 1	1.00 (reference)	0.68 (0.43–1.08)	1.00 (0.65–1.53)	0.87 (0.56–1.34)	0.64 (0.39–1.04)	0.183
Model 2	1.00 (reference)	0.68 (0.43–1.09)	1.01 (0.65–1.57)	0.81 (0.51–1.29)	**0.55 (0.33–0.93)**	0.069
≥65						
Number of participants	186	229	295	275	312	
Number of cases	13	6	13	8	18	
Model 1	1.00 (reference)	**0.35 (0.13–0.95)**	0.59 (0.26–1.33)	**0.39 (0.16–0.98)**	0.84 (0.39–1.82)	0.907
Model 2	1.00 (reference)	**0.32 (0.11–0.89)**	0.48 (0.20–1.13)	**0.33 (0.12–0.87)**	0.74 (0.32–1.72)	0.925
n-6 PUFA, g/day						
<65						
Number of participants	1,203	1,191	1,156	1,134	1,063	
Number of cases	55	37	36	31	30	
Model 1	1.00 (reference)	0.71 (0.46–1.10)	0.72 (0.46–1.13)	0.65 (0.41–1.05)	0.69 (0.42–1.12)	0.113
Model 2	1.00 (reference)	0.72 (0.46–1.13)	0.74 (0.47–1.17)	0.65 (0.40–1.07)	0.68 (0.41–1.14)	0.122
≥65						
Number of participants	205	218	253	275	346	
Number of cases	12	6	17	8	15	
Model 1	1.00 (reference)	0.41 (0.15–1.13)	1.00 (0.44–2.25)	0.41 (0.15–1.10)	0.64 (0.26–1.57)	0.457
Model 2	1.00 (reference)	0.39 (0.14–1.11)	0.95 (0.41–2.21)	0.39 (0.14–1.07)	0.65 (0.25–1.66)	0.489

BMI, body mass index; MUFA, monounsaturated fatty acid; PUFA, polyunsaturated fatty acid, Q, quintile; SFA, saturated fatty acid.

Bold *P* values are statistically significant (*P*<0.05).

The lowest quintiles of total fat and fatty acid intake were used as the reference group in the analysis. Model 1 was adjusted for age and stratified jointly according to areas (Hokkaido, Tohoku, Kanto, Chubu, Kinki, Chugoku, and Kyusyu). Model 2 was additionally adjusted for family history of diabetes (yes, no); family history of hypertension (yes, no); smoking status (never, former smoker, current smoker); body mass index (<18.5, 18.5–24.9, 25.0–30.0, >30.0 kg/m^2); walking hours (almost none, daily 0.5, 0.6–0.9, and ≥1.0 h); hours of exercise (almost none, weekly 1–2, 3–4, and ≥5 h); alcohol consumption habit (never, former drinker, current drinker); energy intake (kcal, continuous); and carbohydrate (g/day; continuous).

[a]Total fat and fatty acid intake were adjusted for energy intake using the nutrient residual model.

[b]The *P* value for linear trend was calculated using a continuous variable of total fat and fatty acid intake assigning the median values in each quartile.

eTable 2. Odds ratios and 95% confidence intervals of type 2 diabetes according to quintiles of energy-adjusted[a] total fat and fatty acid intake by the age (<65 and ≥65 years) among women

	**Q1 (low)**	**Q2**	**Q3**	**Q4**	**Q5 (high)**	***P* for trend**[b]
Total fat, g/day						
<65						
Number of participants	1,878	1,923	2,011	2,024	2,047	
Number of cases	44	39	35	33	40	
Model 1	1.00 (reference)	0.84 (0.54–1.31)	0.72 (0.46–1.14)	0.67 (0.42–1.07)	0.82 (0.52–1.28)	0.254
Model 2	1.00 (reference)	0.83 (0.51–1.35)	0.77 (0.45–1.33)	0.78 (0.43–1.41)	1.01 (0.50–2.05)	0.919
≥65						
Number of participants	530	486	398	385	362	
Number of cases	17	8	12	8	6	
Model 1	1.00 (reference)	0.54 (0.23–1.29)	1.01 (0.46–2.21)	0.70 (0.29–1.69)	0.56 (0.21–1.50)	0.347
Model 2	1.00 (reference)	0.39 (0.15–1.00)	0.66 (0.25–1.71)	0.41 (0.13–1.30)	0.28 (0.06–1.23)	0.121
SFA, g/day						
<65						
Number of participants	1,855	1,936	1,962	2,081	2,049	
Number of cases	38	43	34	40	36	
Model 1	1.00 (reference)	1.09 (0.70–1.70)	0.84 (0.53–1.36)	0.94 (0.59–1.48)	0.83 (0.52–1.34)	0.341
Model 2	1.00 (reference)	1.14 (0.70–1.83)	0.93 (0.55–1.57)	1.11 (0.64–1.92)	1.06 (0.56–2.02)	0.898
≥65						
Number of participants	553	473	447	328	360	
Number of cases	15	15	9	4	8	
Model 1	1.00 (reference)	1.25 (0.59–2.62)	0.78 (0.33–1.85)	0.47 (0.15–1.47)	0.82 (0.33–2.05)	0.317
Model 2	1.00 (reference)	1.08 (0.47–2.44)	1.08 (0.47–2.44)	1.08 (0.47–2.44)	1.08 (0.47–2.44)	0.203
MUFA, g/day						
<65						
Number of participants	1,858	1,949	2,006	2,013	2,057	
Number of cases	42	47	31	35	36	
Model 1	1.00 (reference)	1.08 (0.71–1.66)	0.67 (0.42–1.08)	0.76 (0.48–1.2)	0.78 (0.49–1.24)	0.119
Model 2	1.00 (reference)	0.98 (0.62–1.56)	0.66 (0.39–1.14)	0.74 (0.42–1.31)	0.77 (0.39–1.52)	0.321
≥65						
Number of participants	550	460	403	396	352	
Number of cases	17	8	9	11	6	
Model 1	1.00 (reference)	0.60 (0.25–1.41)	0.77 (0.33–1.78)	0.95 (0.43–2.12)	0.60 (0.23–1.58)	0.493
Model 2	1.00 (reference)	0.45 (0.18–1.16)	0.54 (0.19–1.50)	0.64 (0.21–1.99)	0.36 (0.08–1.66)	0.285

BMI, body mass index; MUFA, monounsaturated fatty acid; PUFA, polyunsaturated fatty acid, Q, quintile; SFA, saturated fatty acid.
Bold *P* values are statistically significant (P<0.05).
The lowest quintiles of total fat and fatty acid intake were used as the reference group in the analysis. Model 1 was adjusted for age and stratified jointly according to areas (Hokkaido, Tohoku, Kanto, Chubu, Kinki, Chugoku, and Kyusyu). Model 2 was additionally adjusted for family history of diabetes (yes, no); family history of hypertension (yes, no); smoking status (never, former smoker, current smoker); body mass index (<18.5, 18.5–24.9, 25.0–30.0, >30.0 kg/m^2); walking hours (almost none, daily 0.5, 0.6–0.9, and ≥1.0 h); hours of exercise (almost none, weekly 1–2, 3–4, and ≥5 h); alcohol consumption habit (never, former drinker, current drinker); energy intake (kcal, continuous); and carbohydrate (g/day; continuous).
[a]Total fat and fatty acid intake were adjusted for energy intake using the nutrient residual model.
[b]The *P* value for linear trend was calculated using a continuous variable of total fat and fatty acid intake assigning the median values in each quartile.

eTable 2. Odds ratios and 95% confidence intervals of type 2 diabetes according to quintiles of energy-adjusted[a] total fat and fatty acid intake by the age (<65 and ≥65 years) among women (continued)

	**Q1 (low)**	**Q2**	**Q3**	**Q4**	**Q5 (high)**	***P* for trend**[b]
PUFA, g/day						
<65						
Number of participants	1,908	2,008	2,004	1,986	1,977	
Number of cases	41	37	30	46	37	
Model 1	1.00 (reference)	0.83 (0.53–1.31)	0.69 (0.43–1.13)	1.09 (0.70–1.7)	0.88 (0.54–1.42)	0.951
Model 2	1.00 (reference)	0.89 (0.55–1.45)	0.8 (0.47–1.36)	1.34 (0.80–2.26)	1.12 (0.60–2.11)	0.384
≥65						
Number of participants	500	401	405	423	432	
Number of cases	13	9	17	8	4	
Model 1	1.00 (reference)	0.92 (0.38–2.23)	1.84 (0.85–3.99)	0.84 (0.32–2.18)	0.40 (0.12–1.37)	0.288
Model 2	1.00 (reference)	0.76 (0.29–1.97)	1.41 (0.58–3.39)	0.53 (0.17–1.64)	0.26 (0.06–1.15)	0.135
n-3 PUFA, g/day						
<65						
Number of participants	1,944	1,997	1,995	1,972	1,975	
Number of cases	39	39	35	45	33	
Model 1	1.00 (reference)	0.97 (0.61–1.52)	0.91 (0.57–1.45)	1.19 (0.76–1.86)	0.84 (0.51–1.36)	0.733
Model 2	1.00 (reference)	0.99 (0.62–1.59)	0.91 (0.55–1.51)	1.24 (0.75–2.05)	0.84 (0.46–1.53)	0.845
≥65						
Number of participants	464	412	414	437	434	
Number of cases	16	5	13	9	8	
Model 1	1.00 (reference)	0.37 (0.13–1.03)	1.05 (0.49–2.25)	0.68 (0.29–1.61)	0.61 (0.25–1.53)	0.461
Model 2	1.00 (reference)	0.32 (0.11–0.93)	0.91 (0.39–2.14)	0.55 (0.21–1.48)	0.49 (0.15–1.60)	0.360
n-6 PUFA, g/day						
<65						
Number of participants	1,901	2,015	2,002	1,986	1,979	
Number of cases	42	37	31	37	44	
Model 1	1.00 (reference)	0.81 (0.51–1.28)	0.69 (0.43–1.13)	0.84 (0.53–1.35)	1.01 (0.63–1.61)	0.863
Model 2	1.00 (reference)	0.9 (0.56–1.45)	0.8 (0.47–1.34)	1.06 (0.63–1.80)	1.33 (0.75–2.37)	0.278
≥65						
Number of participants	507	394	407	423	430	
Number of cases	15	10	12	10	4	
Model 1	1.00 (reference)	0.90 (0.39–2.08)	1.12 (0.49–2.56)	0.89 (0.37–2.16)	0.35 (0.11–1.17)	0.167
Model 2	1.00 (reference)	0.79 (0.32–1.91)	0.84 (0.34–2.07)	0.64 (0.23–1.72)	**0.24 (0.06–0.99)**	0.079

BMI, body mass index; MUFA, monounsaturated fatty acid; PUFA, polyunsaturated fatty acid, Q, quintile; SFA, saturated fatty acid.
Bold *P* values are statistically significant (*P*<0.05).
The lowest quintiles of total fat and fatty acid intake were used as the reference group in the analysis. Model 1 was adjusted for age and stratified jointly according to areas (Hokkaido, Tohoku, Kanto, Chubu, Kinki, Chugoku, and Kyusyu). Model 2 was additionally adjusted for family history of diabetes (yes, no); family history of hypertension (yes, no); smoking status (never, former smoker, current smoker); body mass index (<18.5, 18.5–24.9, 25.0–30.0, >30.0 kg/m^2); walking hours (almost none, daily 0.5, 0.6–0.9, and ≥1.0 h); hours of exercise (almost none, weekly 1–2, 3–4, and ≥5 h); alcohol consumption habit (never, former drinker, current drinker); energy intake (kcal, continuous); and carbohydrate (g/day; continuous).
[a]Total fat and fatty acid intake were adjusted for energy intake using the nutrient residual model.
[b]The *P* value for linear trend was calculated using a continuous variable of total fat and fatty acid intake assigning the median values in each quartile.
